# Associations of subclinical microcalcification and inflammation with carotid atheroma development: a dual-tracer PET/CT study

**DOI:** 10.1007/s00259-025-07127-z

**Published:** 2025-02-13

**Authors:** Shiv Patil, Rithvik Kata, Eric Teichner, Robert Subtirelu, Mohanad Ghonim, Mohamed Ghonim, Omar Al-Daoud, Miraziz Ismoilov, Lancelot Herpin, Cyrus Ayubcha, Thomas Werner, Poul Flemming Høilund-Carlsen, Abass Alavi

**Affiliations:** 1 Department of Radiology, University of Pennsylvania, Philadelphia, PA 19104 USA; 2 Sidney Kimmel Medical College, Thomas Jefferson University, Philadelphia, PA USA; 3 Department of Radiology, Ain Shams University, Cairo, Egypt; 4 Harvard Medical School, Boston, MA USA; 5 Department of Nuclear Medicine, Odense University Hospital, Odense, Denmark

**Keywords:** Carotid artery atherosclerosis, 18F-NaF PET/CT, 18F-FDG PET/CT, Inflammation, Microcalcification

## Abstract

**Purpose:**

Carotid artery atherosclerosis, a significant manifestation of cardiovascular disease (CVD) and leading cause of stroke, develops through a gradual process of arterial inflammation and calcification. This study explores the relationship between arterial inflammation (18 F-FDG PET/CT) and vascular calcification (18 F-NaF PET/CT) in the left and right common carotid arteries (LCC/RCC) and their association with CVD and thromboembolic risk in patients with subclinical atherosclerosis.

**Methods:**

A cohort of 115 subjects (73 healthy volunteers, 42 at-risk for CVD) underwent 18 F-NaF and 18 F-FDG PET/CT imaging. Radiotracer uptake was quantitatively assessed by measuring the average blood-pool-corrected mean standardized uptake value (aSUVmean).

**Results:**

Relative to healthy volunteers, at-risk subjects had greater uptake of NaF and FDG (10–22% and 16–27% higher, respectively, in both arteries, *p* < 0.05). On multivariate regression, NaF aSUVmean correlated with age and BMI (*p* < 0.01), and FDG aSUVmean correlated with BMI (*p* ≤ 0.01), fibrinogen (*p* < 0.01 in LCC only), and total cholesterol (*p* = 0.02 in RCC only). NaF aSUVmean increased with elevated 10-year CVD risk (*p* = 0.003 in LCC only), while no significant trend was seen for FDG. NaF and FDG aSUVmean increased with elevated thromboembolic risk in both arteries (*p* < 0.05). No correlations between NaF and FDG aSUVmean were observed (*p* > 0.05).

**Conclusion:**

18 F-NaF PET/CT may serve as a prognostic tool for carotid microcalcification and subclinical atherosclerosis, while the utility of 18 F-FDG PET/CT remains uncertain.

**Clinical trial registration:**

“Cardiovascular Molecular Calcification Assessed by 18F-NaF PET CT (CAMONA)”, NCT01724749, https://clinicaltrials.gov/study/NCT01724749.

## Introduction

Atherosclerosis is an insidious cardiovascular disease (CVD) that leads to the gradual stenosis of vascular beds over decades, thereby compromising perfusion to vital organs [1]. The progression of atherosclerosis is characterized by a complex pathophysiology of local arterial inflammation and calcification of lipid plaques [2]. Atherosclerosis develops through a gradual process initiated by endothelial cell dysfunction, triggering an inflammatory cascade that recruits macrophages attracted to oxidized lipoproteins in the arterial intima [3, 4]. The persistent aggravation of the arterial wall ultimately leads to tissue hypoxia, plaque necrosis, and structural remodeling via encapsulation of dead macrophages by a thin fibrous cap that is susceptible to rupture [5]. Chronic inflammation of the atherosclerotic plaque promotes mineralization via calcium precipitation, a response that aims to quell inflammation and stabilize the atheroma [6]. These underlying processes are often clinically asymptomatic and progress over an extensive time course causing increased risk of ischemic events such as myocardial infarction and stroke. Early identification of vulnerable patients is therefore critical for effective management and therapeutic intervention [7].

Various imaging modalities such as ultrasonography and computed tomography (CT) are widely used in clinical practice to estimate the severity of atherosclerotic disease [8, 9]. Although these approaches offer detailed resolution of large plaques, their utility in early diagnosis is limited, as macroscopic plaque formation typically represents a relatively late stage of atherosclerosis [10, 11]. This has led to a need to expand imaging modalities beyond the traditional domain of structural anatomy, utilizing the molecular mechanisms that underlie CVD to develop diagnostic methods capable of capturing the subclinical, biologically active stages of atherosclerosis.

Recent advancements in molecular imaging, particularly combined positive emission tomography (PET)/CT, provide new opportunities for assessing early-stage patient vulnerability to atherosclerosis [12, 13]. Two of the most prominent PET radiotracers to emerge in this domain are 18 F-fluorodeoxyglucose (FDG) and 18 F-sodium fluoride (NaF) [14]. As a glucose analog, FDG corresponds to the inflammatory activity of activated macrophages that contribute to plaque formation in vascular beds [15]. NaF uptake is associated with active vascular microcalcification that characterizes unstable or high-risk plaques [16]. In contrast, CT can detect macrocalcification that is reported to be more prevalent in stable plaques that are less likely to rupture [11].

The relationship between CVD risk factors and arterial inflammation and microcalcification has been previously studied in several vascular regions such as the thoracic aorta, coronary arteries, and femoral arteries [17–19]. Analysis of NaF and FDG uptake in the left and right common carotid arteries (LCC / RCC), however, remains limited. Moreover, few molecular imaging studies of atherosclerosis have analyzed this modality for subclinical disease, instead focusing on late-stage, symptomatic patients. In this study, we applied combined PET/CT imaging to evaluate the relationship between CVD risk and carotid artery uptake of both NaF and FDG in a heterogeneous cohort of healthy volunteers and patients at risk for CVD.

## Methods

### Study design

115 subjects were selected from a total of 139 participants enrolled in the Cardiovascular Molecular Calcification Assessed by 18 F-NaF PET/CT (CAMONA) study in Odense, Denmark. Selection for each subject was determined by the quality of PET/CT imaging and feasibility of LCC or RCC segmentation. As such, the exclusion of 24 subjects was a result of incomplete imaging data or an inability to accurately delineate regions of interest (ROIs) for both LCC and RCC due to poor image resolution.

The CAMONA study was approved by the Danish National Committee on Biomedical Research Ethics and is registered on ClinicalTrials.gov under the identifier NCT01724749. This investigation was conducted in agreement with the Declaration of Helsinki and all participants provided written informed consent prior to their participation in the study.

### Subject evaluation

Of the 115 subjects, 73 were healthy volunteers (HVs) and 42 were individuals presenting with symptoms of angina pectoris. HVs were recruited from a sample of Danish citizens without prior history or symptoms of CVD. Patients evaluated for angina pectoris were recruited into the CAMONA study as they were referred for coronary CT angiography. Only patients with a 10-year risk of cardiovascular death greater than or equal to 1% as estimated by the European SCORE system were eligible for inclusion into the at-risk group [20]. Exclusion criteria consisted of the following: pregnancy, malignancy within the past 5 years, known immunodeficiency or autoimmune disease, deep vein thrombosis or pulmonary embolism within the past 3 months, alcohol or illicit drug use, significant mental illness, or initiation of statin therapy within the past 3 months.

All subjects underwent questionnaires, blood testing, and blood pressure measurements. Body height and weight were also recorded to calculate body mass index (BMI). Blood analyses included a lipid profile, fasting plasma glucose, glycated hemoglobin (HbA1c), white blood cell (WBC) count, C-reactive protein (CRP), fibrinogen, homocysteine, and creatinine levels. The Chronic Kidney Disease Epidemiology Collaboration equation was used to estimate glomerular filtration rate (eGFR) as previously described [21]. Blood pressure measurements were taken after subjects rested in a supine position for at least 30 min. A subject was identified as hypertensive if systolic pressure was above 140 mmHg and diastolic pressure was above 90 mmHg or if the subject was currently taking blood pressure lowering medication. The 10-year risk of developing CVD as estimated by the Framingham Risk Score (FRS) and the CHA2DS2-VASc score of thromboembolic risk were measured for all subjects [22, 23]. Additionally, each participant completed questionnaires that assessed smoking history, family history of CVD, and prescription medication.

### PET/CT acquisition protocol

NaF and FDG PET/CT imaging were performed according to published methods [24, 25]. Scans were acquired using hybrid PET/CT systems (GE Discovery STE, VCT, RX, and 690/710 models). Subjects were randomly assigned to a PET/CT system by the hospital department. NaF PET/CT imaging was performed 90 min after administering an intravenous dose of 2.2 MBq of NaF per kilogram of body weight. For FDG PET/CT, subjects were required to fast overnight for at least 8 h and blood glucose concentration was measured to confirm a value below 8 mmol/L. FDG PET/CT imaging was performed 180 min after intravenous injection of 4.0 MBq of FDG per kilogram of body weight. On average, NaF and FDG PET/CT imaging were conducted within two weeks of each other.

PET images were adjusted for factors such as attenuation, scatter, random events or noise, and scanner dead time. Ordered Subset Expectation Maximization (OSEM) approach was used for PET data reconstruction. Low-dose CT scans (140 kV, 30–110 mA, noise rate at 25, 0.8 s for each turn, with a slice thickness of 3.75 mm) were taken for attenuation correction and anatomic co-registration with PET images. The effective radiation dose received from the entire scanning protocol was 14 mSv.

### Quantitative carotid artery PET/CT image analysis

**Fig. 1:**
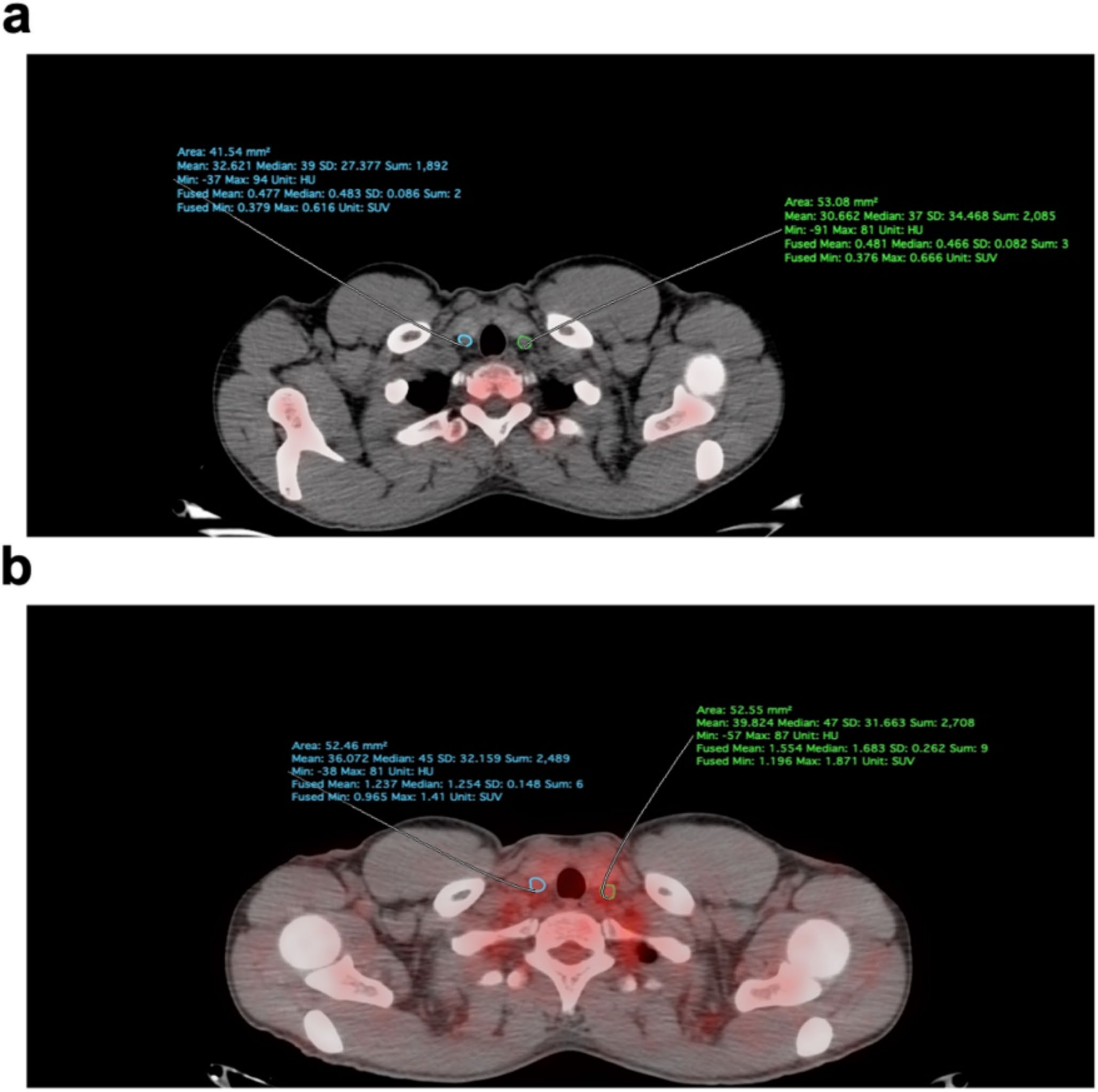
Axial PET/CT image demonstrating manually delineated regions of interest (ROI) around the LCC and RCC to obtain quantitative values (SUVmean, SUVmax) of (**a**) NaF and (**b**) FDG uptake for each artery. Images were analyzed and quantified using OsiriX 7.5.1 software (Pixmeo SARL)

All PET/CT images were analyzed using OsiriX 7.5.1 software (Pixmeo SARL, Bernex, Switzerland). Carotid artery NaF and FDG uptake were quantified as previously described to determine the average mean standardized uptake value (aSUVmean) [24, 25]. In brief, a region of interest (ROI) was manually delineated on the fused PET/CT axial image that surrounded the outer perimeter of either the LCC or RCC. The ROI spanned the whole artery with a slice thickness of 3.75 mm. For each ROI, the mean decay-corrected NaF uptake concentration was calculated. The mean values for each ROI were summed and divided by the total number of slices to calculate the aSUVmean (Fig. 1). Carotid FDG uptake was quantified in a similar manner. To account for potential spillover activity from anatomically adjacent regions, blood NaF activity and blood FDG activity were determined by measuring a ROI in the inferior vena cava. This activity was quantified as the decay-corrected mean radiotracer activity concentration.

While the common carotid arteries are small vessels, the spatial resolution of PET affords reliable global segmentation of this ROI from the origin of the common carotid artery to the point of bifurcation [26, 27]. The ROI-based methodology employed in this study is consistent with that utilized in prior studies of other cardiovascular structures [28, 29].

### Statistical analysis

Subject demographics and baseline clinical characteristics were summarized using descriptive statistics and compared between healthy volunteers and at-risk patients using the independent samples Student’s *t* test, the Mann-Whitney *U* test, or the Fisher’s exact test. Continuous variables were presented as mean ± SD if normally distributed or as median (25th – 75th percentile) otherwise. The 1-sample Kolmogorov-Smirnov test was used to assess normality.

NaF aSUVmean and FDG aSUVmean in both LCC and RCC were adjusted for blood activity, injected radiotracer dose, and PET/CT system using multivariable linear regression as these parameters have been demonstrated to significantly influence quantification of arterial NaF and FDG uptake [30, 31]. The Spearman’s rank correlation coefficient was used to assess the correlation between NaF aSUVmean and FDG aSUVmean. The relationships between cardiovascular risk factors and carotid NaF / FDG aSUVmean were evaluated with univariate linear regression models. A multivariable model was then performed with all predictor variables for which the p-value was less than 0.20. To assess multicollinearity, the variance inflation factor was determined with a threshold of 3.5. The Durbin-Watson test was performed to evaluate the independence of observations, and a normal probability plot was used to check for normality of residuals.

All subjects were stratified into one of three groups based on their 10-year risk of CVD as assessed by FRS: low (< 10%), intermediate (10–20%), or high (> 20%). Analysis of variance (ANOVA) was conducted to compare LCC/RCC NaF aSUVmean and FDG aSUVmean among the three risk groups. Subjects were also stratified based on their CHA2DS2-VASc score into a low (0), moderate (1), or high (2+) thromboembolic risk group, and ANOVA was conducted to assess whether LCC/RCC NaF aSUVmean or FDG aSUVmean significantly differed with increasing thromboembolic risk. Statistical significance was considered at the α = 0.05 level. All analyses were performed using R version 4.0.3.

## Results

The baseline clinical characteristics of the study population are described in Table 1. A total of 115 subjects (age range 21–75 years, 50% male) were included in the analysis. Several differences in subject demographics were observed between HVs and at-risk patients: age, frequency of smoking history, frequency of hypertension, total cholesterol, fasting plasma glucose, HbA1c, median FRS, and median CHA2DS2-VASc score were all significantly greater in the at-risk group. The frequencies of statin and antihypertensive drug use were also higher in the at-risk group. aSUVmean values of NaF and FDG were observed to be greater in at-risk patients (10–22% and 16–27% higher respectively in both arteries, *p* < 0.05). Notably, mean eGFR was lower in the at-risk group than in HVs.

**Table 1:** Baseline clinical characteristics of the study population

	Healthy Volunteers (*n* = 73)	At-Risk Patients (*n* = 42)	*P* Value	Total (*n* = 115)
Age, years	43.1 ± 13.6	56.3 ± 11.6	< 0.01*	47.9 ± 14.4
Male gender, n (%)	38 (52.0)	20 (47.6)	0.79	58 (50.4)
BMI	26.8 ± 4.6	27.0 ± 4.4	0.80	26.9 ± 4.5
Smoking history, n (%)	31 (42.4)	27 (64.2)	0.03*	58 (50.4)
Hypertension, n (%)	6 (8.2)	18 (42.8)	< 0.01*	24 (20.8)
Total cholesterol, mmol/L	4.9 ± 0.8	5.3 ± 0.9	0.01*	5.0 ± 0.9
LDL cholesterol, mmol/L	3.0 ± 0.7	3.3 ± 0.8	0.07	3.1 ± 0.8
HDL cholesterol, mmol/L	1.4 ± 0.4	1.4 ± 0.4	0.83	1.4 ± 0.4
Triglycerides, mmol/L	1.0 ± 0.6	1.1 ± 0.7	0.34	1.1 ± 0.7
Fasting plasma glucose, mmol/L	5.5 ± 0.4	5.8 ± 0.9	0.03*	5.6 ± 0.6
HbA1c (mmol/mol)	33.5 ± 4.1	37.4 ± 5.3	< 0.01*	34.9 ± 4.9
eGFR (mL/min/1.73 m^2^)	83.7 ± 12.8	75.0 ± 14.9	< 0.01*	80.5 ± 14.2
Framingham risk score, % (25–75th percentile)	3.6 (1.6, 8.2)	8.5 (5.8, 16.8)	0.05	7.5 (2.1, 10.3)
CHA2DS2-VASc, % (25–75th percentile)	1.0 (0.0, 1.0)	1.0 (0.0, 2.0)	< 0.01*	1.0 (0.0, 1.0)
Statins	3 (4.1)	14 (33.3)	< 0.01*	17 (14.7)
Antihypertensive drugs	1 (1.3)	18 (42.8)	< 0.01*	19 (16.5)
Injected dose FDG (MBq)	306.7 ± 59.0	308.5 ± 65.3	0.88	307.4 ± 61.1
Injected dose NaF (MBq)	176.4 ± 41.3	172.8 ± 29.1	0.58	175.1 ± 37.2
Circulating time FDG (min)	181.0 ± 3.5	182.1 ± 5.3	0.22	181.4 ± 4.3
Circulating time NaF (min)	91.4 ± 3.9	90.6 ± 3.7	0.26	91.1 ± 3.9
LCC NaF aSUVmean, kBq/mL	0.9 ± 0.2	1.1 ± 0.3	0.04*	0.9 ± 0.2
LCC FDG aSUVmean, kBq/mL	1.1 ± 0.2	1.4 ± 0.4	0.01*	1.2 ± 0.3
RCC NaF aSUVmean, kBq/mL	1.0 ± 0.2	1.1 ± 0.2	0.04*	1.0 ± 0.2
RCC FDG aSUVmean, kBq/mL	1.2 ± 0.2	1.4 ± 0.4	< 0.01*	1.3 ± 0.3

“*” denotes *p* < 0.05

Table 2 lists the univariate correlations between CVD risk factors and uptake of NaF and FDG in the LCC. NaF uptake directly correlated with age, BMI, total cholesterol, triglycerides, HbA1c, and hypertension. FDG uptake was directly associated with age, BMI, total cholesterol, fibrinogen, and hypertension. eGFR inversely correlated with LCC FDG uptake. On multivariate regression, NaF uptake retained direct correlations with age and BMI, while FDG uptake showed direct associations with BMI and fibrinogen (Table 3).

**Table 2:** Univariate correlations between CVD risk factors and NaF / FDG uptake in the LCC

	LCC NaF		LCC FDG	
	Estimate	*P* Value	Estimate	*P* Value
Age	0.005	< 0.01*	0.005	0.01*
Male gender	-0.07	0.11	-0.09	0.16
BMI	0.02	< 0.01*	0.02	< 0.01*
Smoking history	0.04	0.31	0.07	0.28
Total cholesterol	0.06	< 0.01*	0.08	0.03*
HDL cholesterol	-0.02	0.61	-0.02	0.74
LDL cholesterol	0.04	0.10	0.03	0.38
Triglycerides	0.08	0.01*	0.09	0.06
HbA1c	0.01	0.03*	0.01	0.05
eGFR	-0.002	0.10	-0.007	< 0.01*
CRP	0.002	0.78	-0.001	0.90
Fibrinogen	0.004	0.05	0.01	< 0.01*
WBC	0.004	0.70	-0.001	0.91
Hypertension	0.11	0.04*	0.17	0.04*

“*” denotes *p* < 0.05

**Table 3:** Multivariate correlations between CVD risk factors and NaF / FDG uptake in the LCC

	LCC NaF		LCC FDG	
	Estimate	*P* Value	Estimate	*P* Value
Age	0.004	0.02*	-0.001	0.64
Male gender	-0.09	0.06	-0.07	0.33
BMI	0.02	< 0.01*	0.02	0.01*
Total cholesterol	0.04	0.47	0.15	0.09
LDL cholesterol	-0.009	0.88	-0.10	0.28
Triglycerides	0.003	0.92	0.008	0.87
HbA1c	-0.0003	0.95	0.003	0.60
eGFR	0.0005	0.75	-0.004	0.13
Fibrinogen	0.003	0.16	0.01	< 0.01*
Hypertension	0.02	0.63	0.06	0.43

“*” denotes *p* < 0.05

Table 4 lists the univariate correlations between CVD risk factors and uptake of NaF and FDG in the RCC. NaF uptake directly correlated with age, BMI, and fibrinogen. Male gender and eGFR inversely correlated with RCC NaF uptake. FDG uptake was directly associated with age, BMI, total cholesterol, and hypertension. eGFR inversely correlated with RCC FDG uptake. On multivariate regression, NaF uptake retained direct correlations with age and BMI as well as an inverse correlation with male gender, while FDG uptake showed direct associations with BMI and total cholesterol (Table 5).

**Table 4:** Univariate correlations between CVD risk factors and NaF / FDG uptake in the RCC

	RCC NaF		RCC FDG	
	Estimate	*P* Value	Estimate	*P* Value
Age	0.006	< 0.01*	0.006	< 0.01*
Male gender	-0.14	< 0.01*	-0.10	0.13
BMI	0.01	< 0.01*	0.02	< 0.01*
Smoking history	0.05	0.22	0.05	0.42
Total cholesterol	0.04	0.09	0.11	< 0.01*
HDL cholesterol	0.04	0.37	-0.02	0.75
LDL cholesterol	0.009	0.73	0.07	0.07
Triglycerides	0.02	0.40	0.09	0.05
HbA1c	0.007	0.10	0.01	0.09
eGFR	-0.004	0.01*	-0.006	< 0.01*
CRP	-0.001	0.83	-0.002	0.85
Fibrinogen	0.005	0.03*	0.006	0.05
WBC	-0.004	0.74	0.001	0.92
Hypertension	0.11	0.05	0.22	< 0.01*

“*” denotes *p* < 0.05

**Table 5:** Multivariate correlations between CVD risk factors and NaF / FDG uptake in the RCC

	RCC NaF		RCC FDG	
	Estimate	*P* Value	Estimate	*P* Value
Age	0.005	< 0.01*	0.001	0.70
Male gender	-0.14	< 0.01*	-0.09	0.15
BMI	0.01	< 0.01*	0.02	< 0.01*
Total cholesterol	0.009	0.71	0.08	0.02*
Triglycerides	-	-	0.002	0.95
HbA1c	-0.002	0.57	-0.0005	0.94
eGFR	-0.001	0.53	-0.002	0.34
Fibrinogen	0.003	0.16	0.006	0.06
Hypertension	-0.009	0.87	0.11	0.16

“*” denotes *p* < 0.05. Triglycerides were not included in the model for RCC NaF due to a p value exceeding 0.20

**Fig. 2:**
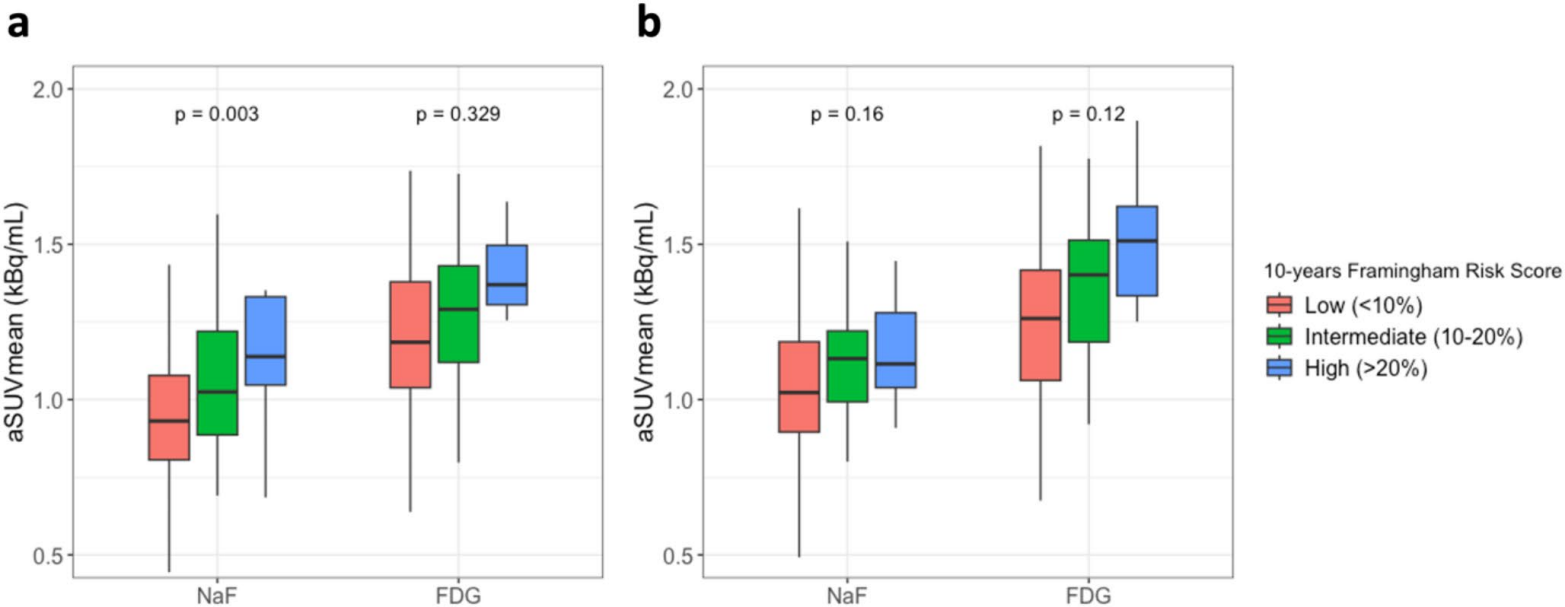
**(a)** Comparison of NaF and FDG aSUVmean in the LCC following stratification of subjects by 10-year FRS. (**b)** Comparison of NaF and FDG aSUVmean in the RCC following stratification of subjects by 10-year FRS

The utility of NaF and FDG PET/CT imaging in the estimation of future CVD risk was assessed by the 10-year FRS (Fig. 2). In the LCC, NaF uptake increased with elevated CVD risk (*p* < 0.01). NaF uptake was on average 29.1% higher in the highest risk group compared to the lowest risk group. FDG uptake was similar in all FRS groups (*p* = 0.32). In the RCC, there were no significant differences in uptake of either NaF or FDG among the three CVD risk groups (*p* = 0.15 and *p* = 0.12, respectively).

**Fig. 3:**
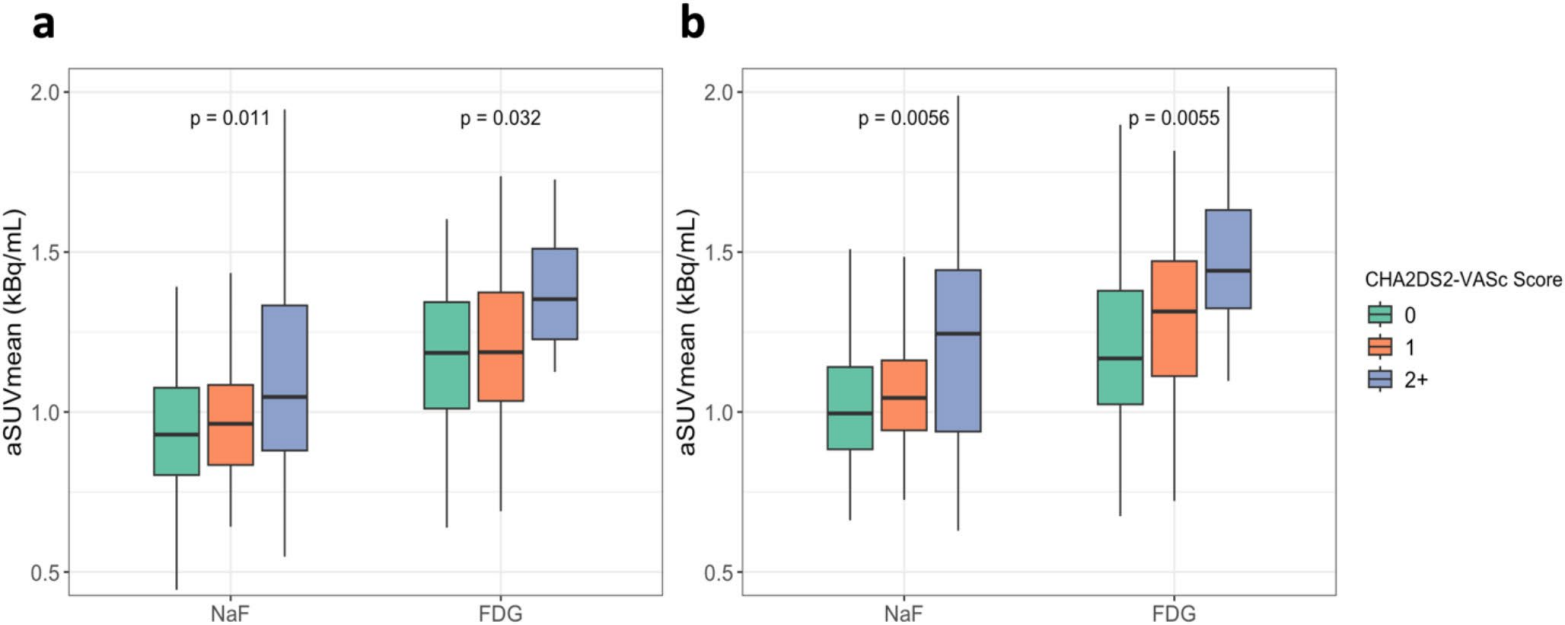
**(a)** Comparison of NaF and FDG aSUVmean in the LCC following stratification of subjects by CHA2DS2-VASc score. (**b)** Comparison of NaF and FDG aSUVmean in the RCC following stratification of subjects by CHA2DS2-VASc score

To assess the utility of NaF and FDG PET/CT imaging in the estimation of future stroke risk, all subjects were stratified based on the CHA2DS2-VASc score into a low (0), moderate (1), or high (2+) thromboembolic risk group (Fig. 3). In the LCC, uptake of both NaF and FDG strongly increased with elevated thromboembolic risk (*p* = 0.01 and *p* = 0.03, respectively). Uptake of NaF and FDG were on average 19.6% and 18.5% higher in the highest risk group compared to the lowest risk group. Similarly, in the RCC, NaF and FDG uptake increased with elevated thromboembolic risk (*p* < 0.01 in both). Uptake of NaF and FDG were on average 19.4% and 21.8% higher in the highest risk group compared to the lowest risk group, respectively.

**Fig. 4:**
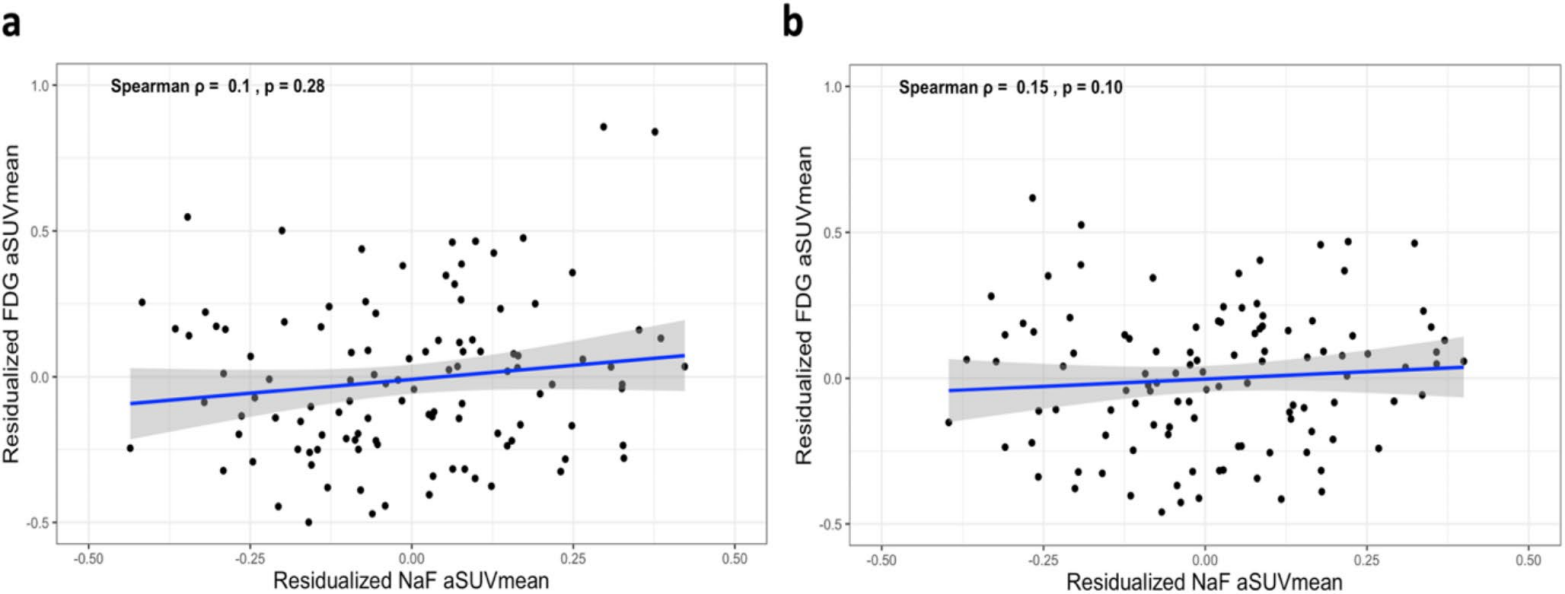
No correlation was observed between NaF aSUVmean and FDG aSUVmean in either the **(a)** LCC or (**b)** RCC after adjusting for patient age and gender

The relationship between carotid artery microcalcification and inflammation was assessed by Spearman’s rank correlation. In both LCC and RCC, while a strong direct correlation between NaF and FDG uptake was initially observed, this correlation did not retain statistical significance after adjusting for patient age and gender (Fig. 4).

## Discussion

In the present study, we quantified microcalcification and inflammation with NaF and FDG PET/CT to investigate the use of these radiotracers in evaluating carotid atheroma development. We found that bilateral carotid artery uptake of NaF and FDG were higher in patients at risk for CVD relative to HVs and correlated with several established atherogenic risk factors including age, BMI, hypercholesterolemia, and hypertension. Linear regression models of NaF and FDG uptake revealed no significant correlations between the two tracers after adjusting for patient age and gender. In addition, while both NaF and FDG were associated with an elevated predicted risk of thromboembolism as estimated by the CHA2DS2-VASc score, only NaF correlated with increased risk of adverse cardiovascular events as assessed by the 10-year FRS.

Several studies have examined NaF and FDG PET/CT imaging of atherosclerotic disease. In an analysis of 269 oncologic patients, Derlin et al. observed correlations between carotid artery NaF uptake and numerous CVD risk factors such as age, male sex, hypertension, and hypercholesterolemia in 296 oncologic patients [32]. Lee et al. quantified carotid artery FDG uptake in 290 asymptomatic adults, reporting associations between FDG and triglyceride levels, diabetes, and metabolic syndrome [33]. Using a dual-tracer approach, Blomberg et al. found that uptake of NaF, but not FDG, in the thoracic aorta of 139 subjects from the CAMONA trial correlated with increased risk of CVD [28]. In the abdominal aorta, Arani et al. identified associations between NaF and age as well as the 10-year FRS in 123 CAMONA subjects, while no associations with FDG were demonstrated [34]. Overall, the findings obtained in our study are largely consistent with those reported in the literature.

The predictive utility of NaF and FDG PET for stroke has also been previously reported. In a study of 40 at-risk patients from the CAMONA trial, Gonuguntla et al. found that global cardiac NaF uptake directly correlated CHA2DS2-VASc score [35]. Castro et al. observed a direct association between LCC NaF uptake and both FRS and CHA2DS-VASc score in 128 subjects from the CAMONA trial [36]. In a study of 230 patients (115 with atrial fibrillation), Wang et al. concluded that right atrial FDG uptake is an independent risk factor for stroke and can improve the predictive performance of the CHAD2S2-VASc score [37]. The results from our study further support these conclusions by demonstrating a relationship between stroke risk and uptake of NaF and FDG in both left and right common carotid arteries.

Our findings indicate that certain associations of NaF or FDG with cardiovascular risk factors are laterality-specific, or seen for one carotid artery but not for the other side. This is consistent with various studies that have observed differences in left and right carotid plaque composition and vulnerability [38–40].The unique anatomical configuration of each vessel, with the LCC originating directly off the aortic arch and RCC branching from the brachiocephalic artery, may cause distinct susceptibility to hemodynamic shear stress and atherosclerotic risk factors. For instance, Stevens et al. reported that right carotid intima-media thickness (IMT) was correlated with hemodynamic parameters (e.g., hypertension), while left IMT was more strongly linked to metabolic indices [41]. The results from our study also show univariate direct correlations of LCC microcalcification with metabolic factors (total cholesterol, triglycerides, and HbA1c) that are not found in the RCC. However, hypertension was associated with microcalcification in the LCC but not in the RCC.

The pathophysiology of atherosclerosis is highly amenable to visualization by PET, which enables the detection of molecular mechanisms that precede the development of macroscopic plaques visible on CT or structural MRI [42]. Early stages of calcification involve cellular-level plaque microcalcifications that actually increase local tissue stress and exacerbate the risk of rupture [43]. Advanced atherosclerosis is characterized by the formation of stable macrocalcifications, which can further the risk of adverse cardiovascular events as a result of arterial stenosis [44]. The presence of vascular calcification is therefore associated with overall atherosclerotic disease vulnerability irrespective of plaque stability [45]. As a glucose analog, FDG uptake reflects the increased metabolic activity of the inflammatory state driven by macrophages localized to the atheroma [46]. NaF retention in the arterial wall corresponds to the adsorption of hydroxyapatite crystals exposed in areas of active microcalcification [47]. Early studies described an association of subclinical arterial calcification with inflammation, thus serving as a potential therapeutic target for CVD together with the anti-inflammatory action of statins [48, 49]. In contrast, while our study demonstrated that carotid uptake of both NaF and FDG correlated with numerous atherosclerotic risk factors, we found no significant association between these two radiotracers. This is consistent with prior studies that report only minor colocalization of NaF and FDG in arterial plaques [18, 28, 50]. The lack of association between NaF and FDG suggests the two radiotracers may represent distinct stages of atherosclerosis: initial plaque formation driven by inflammatory cells (FDG) followed by active plaque mineralization (NaF) in progressive disease [11, 51, 52].

Our study also indicates that NaF PET/CT may have prognostic value in evaluating risk of CVD and thromboembolism for patients with subclinical atherosclerosis. We found that FDG PET/CT, however, has limited use in assessing CVD risk. One possible explanation for this is that arterial inflammation is a significant feature of both early and advanced atherosclerosis [53]. As such, FDG uptake by retained macrophages cannot accurately assess plaque vulnerability, and thus poorly correlates with the 10-year FRS. In contrast, NaF uptake is more specific to high-risk vulnerable plaque formation [47].

This study assesses NaF and FDG PET/CT imaging of both left and right common carotid arteries in a heterogenous population of patients with low-to-moderate risk of CVD, extending findings observed in prior studies of patients with late-stage or symptomatic CVD [54–57]. For instance, Quirce et al. evaluated NaF and FDG uptake in carotid plaques from 9 patients investigated for recent stroke, reporting that active calcification is predominant in both the symptomatic and asymptomatic atheroma [55]. Vesey et al. performed NaF and FDG PET/CT in 26 patients after recent transient ischemic attack (TIA) or stroke and concluded that NaF is a superior radiotracer of culprit and phenotypically high-risk carotid plaque [56]. Joshi et al. also demonstrated significant NaF uptake at the site of plaque rupture that correlated with histological features (active calcification, macrophage infiltration, apoptosis, and necrosis) in 12 patients undergoing carotid endarterectomy for symptomatic carotid artery disease [57]. Our study corroborates these findings and reveals the prognostic utility of NaF PET/CT in healthy volunteers and patients with subclinical atherosclerosis prior to the onset of symptomatic manifestations such as TIA or stroke. While further longitudinal studies are needed, these results demonstrate the promising clinical role that NaF PET/CT may have as a screening tool of early-stage atherosclerosis. The inclusion of a patient population with a wide age range (21–75) further affords our study a comprehensive assessment of the relationship between microcalcification and inflammation with the chronic timeline of atherosclerotic disease progression.

The findings of this study, however, should be interpreted within the bounds of certain limitations. First, the risk of CVD and thromboembolism were calculated using FRS and CHA2DS2-VASC scores respectively, which are susceptible to under- or over-estimation [58, 59]. Second, the relationship between atheroma development and vascular inflammation and microcalcification were evaluated using a cross-sectional design. Identifying the association of baseline carotid NaF or FDG uptake with follow-up data on patient outcomes (e.g., development of TIA or stroke) would provide greater validation of prognostic utility compared to risk stratification scores. Future studies should implement longitudinal design to evaluate temporal relationships between NaF/FDG PET and carotid atherosclerosis. Third, several subjects in this study were under statin therapy (17/115), which has been shown to reduce plaque retention of both FDG and NaF [49, 60]. While subjects who had begun statin therapy within 3 months of the CAMONA trial were excluded from our analysis, the long-term effects of such treatment may influence the findings we observed between NaF and FDG uptake. Fourth, manual segmentation was used to quantify tracer uptake in each axial PET/CT slice, which may introduce technical error. The use of artificial intelligence (AI)-based methods to automatically segment arterial structures is a promising advancement that may minimize error produced by manual techniques, yet may require the acquisition of higher quality scans [61].

## Conclusion

This study evaluated the association of vascular microcalcification and inflammation with carotid atheroma development based on 18 F-NaF and 18 F-FDG PET/CT. Our findings support NaF PET/CT imaging in identifying patients with an elevated risk of CVD and thromboembolism, whereas the utility of FDG PET/CT in this domain is less clear. NaF PET/CT is a promising modality for the detection of subclinical carotid artery atherosclerosis and high-risk atheroma development, providing an avenue for earlier prognostication and therapeutic intervention. Future longitudinal studies with NaF and FDG PET/CT are required to assess the temporal relationship of carotid artery microcalcification and inflammation with risk of CVD and thromboembolism.

## Data Availability

The datasets analyzed during the current study are available from the corresponding author on reasonable request.
